# Centrifugally Spun PVA/PVP Based B, N, F Doped Carbon Nanofiber Electrodes for Sodium Ion Batteries

**DOI:** 10.3390/polym14245541

**Published:** 2022-12-18

**Authors:** Meltem Yanilmaz, Elham Abdolrazzaghian, Lei Chen, Juran Kim, Jung Joong Kim

**Affiliations:** 1 Department of Nano Science and Nano Engineering, Istanbul Technical University, Istanbul 34469, Turkey; 2 Department of Textile Engineering, Istanbul Technical University, Istanbul 34469, Turkey; 3 School of Textile Science and Engineering, Tiangong University, Tianjin 300387, China; 4 Advanced Textile R&D Department, Korea Institute of Industrial Technology (KITECH), Ansan 15588, Republic of Korea; 5 Department of Civil Engineering, Kyungnam University, Changwon 51767, Republic of Korea

**Keywords:** centrifugal spinning, carbon nanofiber, PVA, PVP

## Abstract

Owing to their high electrical conductivity, high surface area, low density, high thermal stability, and chemical stability, carbon nanofibers have been used in many fields, including energy storage, electromagnetic shielding, filtering, composites, sensors, and tissue engineering. Considering the environmental impact of petroleum-based polymers, it is vital to fabricate carbon nanofibers from environmentally-friendly materials using fast and safe techniques. PVA/PVP nanofibers were fabricated via centrifugal spinning and the effects of variations in the PVP content on the morphology and thermal properties of PVA/PVP-blend nanofibers were studied using SEM and DSC analyses. Moreover, the effects of carbonization conditions, including stabilization time, stabilization temperature, carbonization time, and carbonization temperature on the morphology and carbon yield, were investigated. Centrifugally spun PVA/PVP-based carbon nanofiber electrodes with an average fiber diameter around 300 nm are reported here for the first time. Furthermore, centrifugally spun PVA/PVP-based B, N, F-doped carbon nanofibers were fabricated by combining centrifugal spinning and heat treatment. Through B, N, F doping, CNFs demonstrated a high reversible capacity of more than 150 mAh/g in 200 cycles with stable cycling performance.

## 1. Introduction

Rechargeable batteries have attracted great attention as reliable and environmentally friendly energy storage devices due to the increasing effects of global warming and the increasing usage of fossil fuels. Rechargeable batteries, which use electrochemical energy to produce electricity, are used in many diverse fields, including electric vehicles, hybrid cars, portable electronics, etc. [1]. Lithium-ion batteries (LIBs) have high energy density, high cycling stability, low weight, and good rate capability and thus they are one of the most commonly used types of rechargeable batteries [2]. However, due to their high cost and limited lithium resources, sodium-ion batteries (SIBs) have been introduced as promising energy storage systems due to the abundance and low cost of sodium resources [3].

In rechargeable batteries, choosing an appropriate anode that can accommodate a greater number of ions and exhibit high electronic conductivity is very important [4]. Carbon-based materials such as graphite, graphene, carbon nanofibers (CNFs), carbon nanotubes, carbon nanospheres, carbon nanowires, hard carbons, and soft carbons, have been used as anodes due to their abundance, low cost, and good conductivity [5]. CNFs with excellent conductivity are considered one of the most promising anode materials in rechargeable batteries [6]. CNFs with a one-dimensional (1D) structure are composed of disordered graphite layers, which provide a large surface-to-volume ratio and a short distance for the transfer of ions [7]. They have diverse applications and are used in many fields, including energy storage technologies, sensors, tissue engineering, drug delivery, and membranes [8]. CNFs are synthesized via different techniques, such as chemical vapor deposition, electrospinning, and centrifugal spinning [9]. Among these methods, electrospinning is the most popular method. However, this method has some disadvantages such as a low production rate, safety issues, and the consumption of a large amount of solvents. Carbon nanofibers can also be produced via centrifugal spinning and this technique has the merits of a high production rate, simplicity, environmental friendliness, and low cost [10,11,12,13,14,15,16,17].

Polyacrylonitrile (PAN) is prepared via the polymerization of acrylonitrile and it is the most commonly used petroleum-based polymer in the production of carbon nanofibers due to its high carbon yield. However, the disadvantages of its high cost and shrinkage after drying limit its usage [18,19]. Moreover, considering the environmental impact of petroleum-based polymers, it is vital to fabricate carbon nanofibers from environmentally-friendly materials using fast and safe techniques [20,21]. Polyvinyl alcohol (PVA) and polyvinyl pyrrolidone (PVP) are hydrophilic polymers that can be used to produce CNFs. PVA (C_2_H_4_O)n) is a hydrophilic and semi-crystalline polymer composed of vinyl monomer units that has a repeating hydroxyl group (OH) in its unit [22]. Interconnected hydrogen bonding makes this polymer cross-linkable. Because of these properties, PVA fibers have shown good mechanical strength and high chemical resistance. Furthermore, this polymer is non-toxic, biodegradable, and biocompatible [23]. Due to the easy breakage of the hydroxyl groups of PVA when it is heated, it is a good precursor material for the production of carbonaceous material [24]. During carbonization, the OH group, the CO group (carbonyl group), and non-carbon elements disappear, so the carbon content increases as the temperature increases steadily [25]. (PVP), (C6H9NO)n is a hydrophilic polymer composed of linear 1-vinyl-2-pyrrolidinone groups [26]. It is a non-ionic polymer that has many applications in diverse fields due to its good solubility in water and many organic solvents, its superior chemical and thermal stability, its non-toxicity, and its affinity for both hydrophobic and hydrophilic materials [27].

Heteroatom-doped carbon materials have attracted wide interest due to their impressive performance in diverse applications. Heteroatom doping is one of the most efficient ways to enhance the performance of carbon-based materials. In this process, heteroatoms, such as nitrogen, fluorine, phosphorous, boron, sulfur, etc., are introduced into carbon materials. Doping alters the band gap of the carbon matrix, which leads to faster electron transfers and the destruction of electroneutrality. For these reasons, the physical and chemical properties of carbon, such as the amount of defects, the interlayer space, the electrical conductivity, and the number of active sites can be improved, and this leads to enhancements in electrochemical performance, specific capacity, and energy density [28,29].

Some studies have reported on PVA- and PVP-based carbon nanofibers obtained via electrospinning. However, no systematic study has been conducted on the effect of carbonization conditions on the morphology and carbon yield of PVA-based centrifugally spun carbon nanofibers. In this study, PVA, PVP, and PVA/PVP-blend nanofibers were produced via centrifugal spinning. Parameter optimization for PVA was performed and the effect of PVP content on fiber morphology and thermal properties was investigated. PVA/PVP-blend fibers with 25% PVP were used to obtain carbon nanofibers. Centrifugally spun PVA/PVP-based CNFs with an average fiber diameter around 300 nm were prepared and the effect of carbonization conditions on the morphology and carbon yield was investigated for the first time. The obtained carbon nanofibers were used as anodes in sodium ion batteries. In order to further improve their electrochemical properties, centrifugally spun PVA/PVP-based B, N, F-doped carbon nanofibers were prepared. We fabricated centrifugally spun PVA/PVP-based B, N, F-doped carbon nanofibers with an average fiber diameter of around 300 nm and observed their excellent cycling stability with a high capacity in Na-ion batteries.

## 2. Materials and Methods

Poly (vinyl alcohol) (PVA) with an average Mw of 85,000–124,000 (87%–89% hydrolyzed), polyvinylpyrrolidone (PVP) with an average Mw of ~1,300,000, and 1-Butyl-3-methylimidazolium tetrafluoroborate (≥98%) and ethanol were purchased from Sigma-Aldrich Gillingham, UK. Ethanol and distilled water were used as solvents.

In this study, 10 wt. %, 13 wt. %, and 15 wt. % PVA solutions were prepared by dissolving PVA in 10 mL of distilled water under magnetic stirring at 70 °C. Furthermore, a 10 wt. % PVP solution was prepared via the dissolution of 1 g polymer powder in ethanol/distilled water under constant stirring for 24 h. Additionally, 15 wt. % PVA and 10 wt. % PVP were mixed in ratios of 25/75, 50/50, and 75/25 to prepare PVA/PVP-blend solutions (Table 1). All solutions are fed into the machine with the aid of a syringe pump at a speed of 60 mL/h. The speed of the spinneret varied between 2000 to 5000 rpm. The distance between the collector and the spinneret was 10 cm and the nozzle diameter used in this process was 0.5 mm.

PVA/PVP-blend nanofibers were carbonized at varying stabilization temperatures, times, and carbonization temperatures. PVA/PVP-blend fibers were stabilized at 150 °C and 280 °C in air and carbonized at 650 °C and 800 °C in N_2_. The effect of varying stabilization times (0, 2, 8, 24 h) on carbon content was also investigated (Table 2).

**Figure 1:**
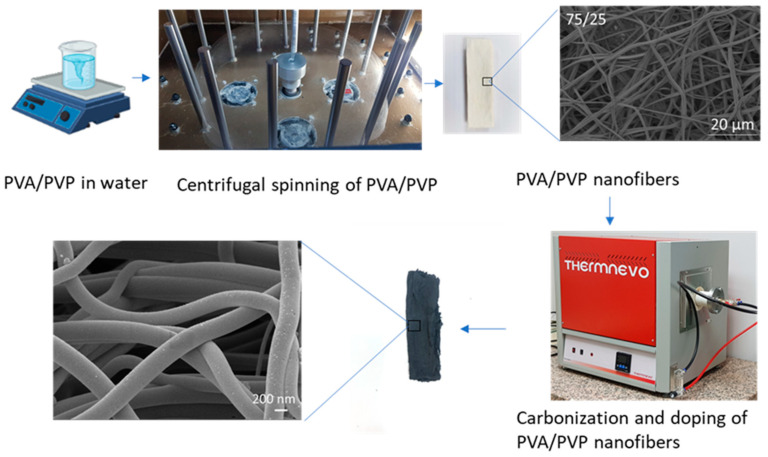
Schematic illustration of the preparation of centrifugally spun B, N, F-doped carbon nanofibers.

In order to produce boron, nitrogen, and fluorine (B, N, F)-doped PVA/PVP-based carbon nanofibers, 0.5 mL 1-butyl-3-methylimidazolium tetrafluoroborate was dissolved in 5 mL of distilled water; then, the PVA/PVP-based carbon nanofibers were immersed in this solution. After drying, PVA/PVP-based carbon nanofibers were put in a furnace and heated to 700 °C with a heating rate of 10 °C under a nitrogen atmosphere and held at 700 °C for 10 h. A schematic illustration of the preparation of centrifugally spun B, N, F-doped CNFs is shown in Figure 1.

SEM images of the precursor fibers were obtained to investigate the morphologies and diameters of the fibers. Differential scanning calorimetry (DSC) was used to analyze the physical transformation behavior of the material under a heating rate of 10 °C/min in a nitrogen atmosphere. XRD and Raman spectroscopy were used to characterize the carbon nanofiber structure. Electrochemical experiments were performed using two electrode coin-type (316ss CR2032) half cells. A Whatman glass microfiber filter membrane was used as a separator and sodium metal plates were used as the counter electrodes. Electrolyte of the sodium ion battery was composed of 1.0 M of NaClO_4_ in ethylene carbonate (EC) and propylene carbonate (PC) with a 1:1 volume. Galvanostatic charge/discharge measurements were performed to investigate the cycling stability of the material. A constant current was applied to the working electrode between two potential values of 0–2.5 V. The rate capabilities for all electrodes were evaluated at high current densities ranging from 0.1 A/g to 2 A/g at room temperature.

## 3. Results

### 3.1. Optimization for PVA Nanofibers

**Figure 2:**
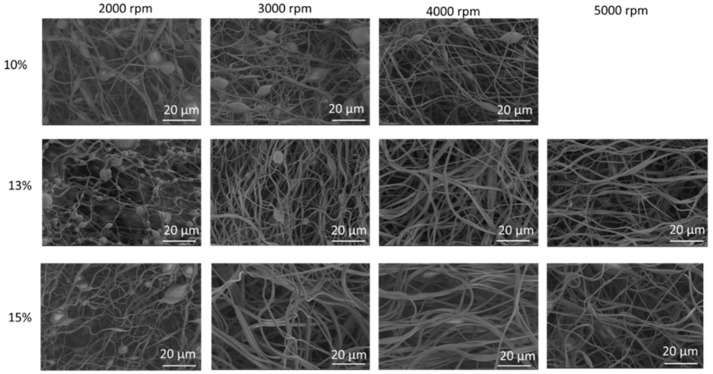
SEM images of PVA nanofibers fabricated with different solution concentrations and rotation speeds.

PVA, PVP, and PVA/PVP-blend nanofibers were synthesized via centrifugal spinning. During fiber production, the polymer solution is fed into the spinneret via a syringe pump. When the speed reaches a critical value, the centrifugal force overcomes the surface tension and then a polymer jet comes out through the nozzles. Centrifugal force stretches the liquid jet, which leads to nanofiber formation. The morphology of the nanofibers can be affected by different factors, such as the nozzle diameter, rotation speed, flow rate, temperature, spinneret-to-collector distance, viscosity, concentration, and the surface tension of the solution [12,13,30]. Viscosity is one of the most important factors that directly affect fiber formation and the concentration of the spinning solution. If the viscosity of the solution is too high, the fibers cannot be produced due to the large gravitation between molecules and the lack of sufficient strength to stretch the liquid jet. If the viscosity is too small, droplets are produced instead of nanofibers during the process [31]. The concentration and rotational speed are also important parameters in fiber formation and in relation to the average fiber diameter [13,30]. PVA nanofibers were prepared using three different concentrations, 10%, 13%, and 15%, and four different rotational speeds, 2000, 3000, 4000, and 5000 rpm. Figure 2 displays SEM images of PVA nanofibers obtained with varying concentrations and rotational speeds. The viscosities of PVA nanofibers obtained with 10%, 13%, and 15% concentrations were 104, 239, and 305 Pa·s at 10 rpm (Table 1). Increasing concentrations led to increased viscosities and larger average fiber diameters for all studied rotational speeds. The average fiber diameters were 613, 615, and 790 nm for PVA nanofibers produced using 10%, 13%, and 15% solutions at 4000 rpm, respectively. The rotational speed plays an important role in fiber formation as well. If the rotational speed is too low, the centrifugal force cannot overcome the surface tension to produce nanofibers. As seen in the SEM images, increasing speed led to decreased average fiber diameters for all studied concentrations. For the 10 wt. % PVA solution, PVA nanofibers had average diameters of 613, 550, and 549 nm, respectively, at 2000, 3000, and 4000 rpm. Bead formation was seen at 2000, 3000, and 4000 rpm when the concentration was 10% due to insufficient polymer chain entanglement and no fiber formation was observed when the rotational speed was increased to 5000 rpm. When the concentration of the solution does not reach a critical value, the chain entanglements become insufficient, thus leading to the formation of beads during fiber spinning [10]. PVA nanofibers fabricated using the 13% solution had average diameters of 615, 573, 570, and 569 nm, respectively, at 2000, 3000, 4000, and 5000 rpm. When the liquid jet was ejected from the nozzles toward the collectors, the speed of the liquid jet decreased steadily. When the spinneret rotated at a higher speed, the speed of the liquid jet became higher and the centrifugal force and air force increased, which led to the formation of fibers with smaller diameters. Similar results were reported by Atici et al. [13], Lu et al. [10], and Chen et al. [10]. Moreover, the average fiber diameters were 790, 763, 690, and 571 nm for the PVA nanofibers produced using the 15% solution, respectively, at 2000, 3000, 4000, and 5000 rpm. At 2000 rpm, bead formation was observed at all studied concentrations. As the rotational speed increased, the average fiber diameter of the PVA nanofibers decreased and led to the formation of long, smooth nanofibers without beads. Lu et al. [10] studied the effect of rotational speed and observed that with increasing rotating speeds from 2000 to 4000 rpm, the average diameter of nanofibers decreased from 663 to 440 nm due to the increasing centrifugal force applied to the solution.

### 3.2. PVA/PVP Nanofibers with Different PVP Ratios

**Figure 3:**
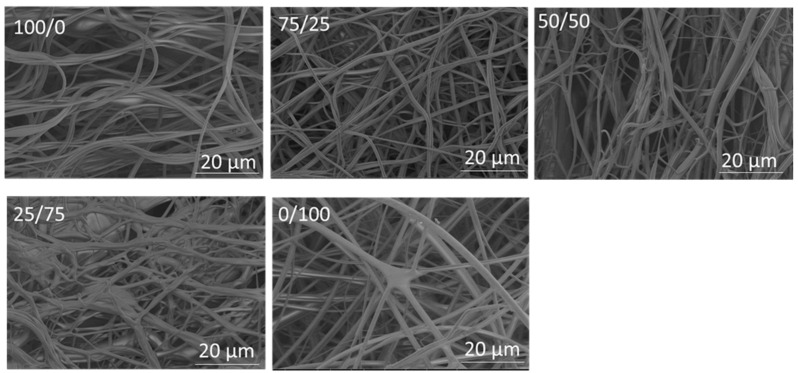
SEM images of PVA, PVP, and PVA/PVP nanofibers with different PVA/PVP ratios.

Electrospun PVA/PVP-blend nanofibers have been used in many fields, including but not limited to biomaterials, drug delivery, scaffolds, dressings, sensors, and packaging [32,33,34]. PVP improves the spinnability as well as the applicability of PVA [35]. Moreover, electrospun PVA/PVP-blend nanofibers bring about a sustained release of drugs, along with good biocompatibility and biodegradability in bio-pharmaceutics [32]. Considering the drawbacks of electrospinning, it is important to fabricate PVA/PVP nanofibers via a fast, safe and more environment-friendly technique. PVA/PVP nanofibers were fabricated here via centrifugal spinning. In order to produce PVA/PVP-blend nanofibers, 15 wt. % PVA and 10 wt. % PVP solutions were blended together in the ratios of 75/25, 50/50, and 25/75 PVA and PVP, respectively. Figure 3 shows SEM images of PVA/PVP-blend nanofibers with different ratios. SEM images of PVA and PVP nanofibers are also shown for comparison. The average diameters of PVA/PVP nanofibers with the ratios of 75/25, 50/50, and 25/75 were 738, 747, and 1093 nm, respectively. The viscosities were 361, 306, and 254 Pa·s for PVA/PVP-blend solutions with 75/25, 50/50, and 25/75 ratios of PVA and PVP, respectively. The average diameters of the nanofibers increased as the amount of PVP increased in the solution. As shown in Figure 3, the use of a PVA/PVP ratio of 75/25 led to the formation of long fibrous smooth nanofibers without bead formation; however, when the ratio of the PVA/PVP solution was changed to 50/50 and 25/75, the morphology of the nanofibers started to change and long fibers were stuck together, which resulted in increased average fiber diameters.

**Figure 4:**
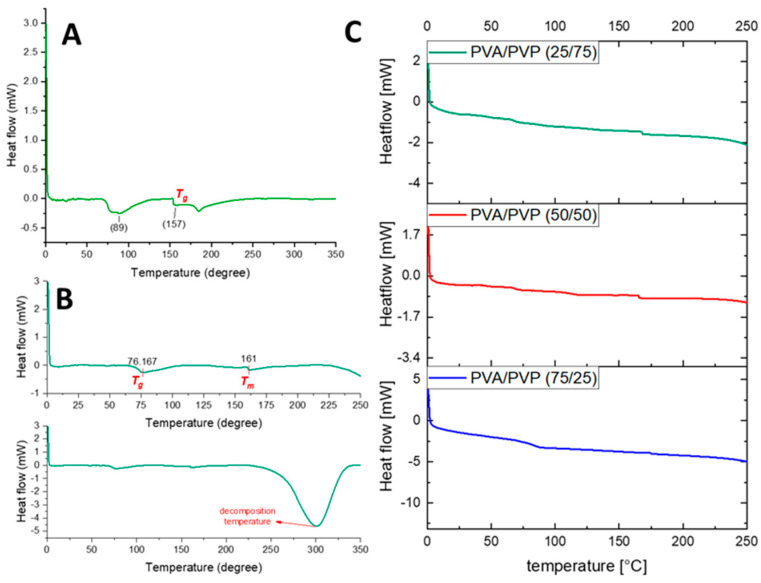
DSC thermograms of (**A**) PVP, (**B**) PVA, and (**C**) PVA/PVP blend nanofibers.

Differential scanning calorimetry (DSC) was used to analyze the physical transformation behavior of the material at a heating rate of 10 °C/min in a nitrogen atmosphere. The glass transition temperature (*Tg*), and melting temperature (Tm) are important pieces of information that can be observed during this process [36]. About 2 mg of each sample was sealed in aluminum pans and heated from 0 °C to 250 °C to clear any thermal history, then cooled to 0 °C for crystallization. Afterwards, the samples were heated again from 0 °C to 350 °C to obtain DSC endotherms. Figure 4 shows the thermograms of the PVA and PVP nanofibers. Figure 4A presents the DSC curve of the PVP nanofibers, which shows a large endothermic peak at 89 °C (Tg), related to the water evaporation of PVP nanofibers [37]. Moreover, some studies have reported glass transition temperatures between 86 °C and 178 °C for PVP [38]. The peak at 157 °C revealed the glass transition temperature (Tg). The different values of Tg reported in these studies are related to the diverse molecular weights of PVP and varying preparation situations [39]. As shown in Figure 4B, the first endothermic peak observed at 76 °C was attributed to the glass transition temperature (Tg) of PVA nanofibers [40]. The second endothermic peak, observed at about 160 °C, corresponded to the melting point of PVA, which revealed its crystalline characteristics [41]. The large peak observed at 300 °C may be attributed to the decomposition of PVA [42,43,44].

### 3.3. Carbonization of PVA/PVP Nanofibers

After centrifugal spinning, PVA/PVP nanofibers were converted to CNFs by applying thermal treatment that included stabilization and carbonization. Eight different heat treatments with varying stabilization and carbonization temperatures and times were studied. Heat treatment results are presented in Table 2. The effect of different heat treatment conditions, including stabilization at 150 °C and 280 °C and carbonization at 600 °C and 800 °C, with times varying from 2 h to 24 h were investigated. During carbonization, when PVA is heated at higher temperatures than its melting point, a low carbon yield will be obtained due to decomposition [45]. Stabilization is carried out to prevent PVA from melting at high temperatures before carbonization [46]. The stabilization process was performed under air atmosphere, which means that air could circulate in the oven to import oxygen into the nanofibers and remove exothermic heat, thus preventing PVA nanofibers from melting at high temperature [47]. In the stabilization process, water evaporates from the structure and a polyene-type structure is generated. Furthermore, the hydroxyl groups present in the molecular chain of PVA dehydrated to C-O and C=O, while preserving C-C, leading to an increased number of cyclization reactions and an enhanced carbon yield obtained after carbonization. In the carbonization process, most of the non-carbon elements assume their gaseous form and are removed from the structure and only carbon atoms remain [48].

A stabilization step is also necessary in the carbonization process of PVP nanofibers in order to prevent the thermoplastic polymer from melting and to preserve the fibrous structure of nanofibers. While diffusing oxygen in the stabilization process, many oxygen-containing functional groups are produced on the aromatic rings. During this step, PVP undergoes cyclization and crosslinking reactions [49]. The oxygen that is present in the air chemisorbs into the carbon surface and carbon-oxygen groups are formed. Furthermore, the color of the nanofibers changes to yellow due to increased carbon content [8]. During stabilization, several chemical reactions, such as crosslinking, oxidation, and dehydrogenation occur, whereas during carbonization only dehydrogenation occurs under a nitrogen atmosphere. During carbonization, as temperature increases, the linkages of polymer chains start to break (C-N bonds) and carbon groups (C-C bonds) remain in the structure, so carbon material is produced [47,50,51].

In the first process (PVA/PVP-1), stabilization was conducted at 150 °C for 24 h and at 280 °C for 6 h and then stabilized PVA/PVP nanofibers were carbonized at 800 °C. The carbon yield was 6.02%; however, when the carbonization temperature was 650 °C (PVA/PVP-3), the carbon yield increased to 19.69%, which shows that increasing the carbonization temperature decreased the yield. When no stabilization process was applied (PVA/PVP-2), the carbon yield was only 11.61%. As stabilization was performed only at 280 °C, followed by carbonization at 650 °C (PVA/PVP-4), the carbon yield was 19.00%, which was close to that of PVA/PVP-3. When nanofibers were stabilized at 150 °C for 24 h, followed by carbonization at 650 °C (PVA/PVP-5), the carbon yield was 15.93%, which proved that stabilization at 280 °C was effective in improving the carbon yield. As nanofibers stabilized at 150 °C and 280 °C for 2 h, followed by carbonization at 650 °C (PVA/PVP-6), the carbon yield was 5.80%, which showed that the duration of 2 h was not sufficient for stabilization, leading to a low carbon yield. When stabilization was performed at 280 °C for 2.5 h, followed by carbonization at a higher temperature of 800 °C (PVA/PVP-7), the carbon yield decreased to 4.78%. When nanofibers underwent stabilization processes at 50 °C, 150 °C, and 220 °C, followed by carbonization at 50 °C, 220 °C, 250 °C, and 800 °C (PVA/PVP-8), a carbon yield of 27.16 was observed even at high carbonization temperatures, which proved that stabilization conditions had an effect on carbon yield. During this process, the carbon yield was the highest compared to all conditions owing to the use of a sufficient stabilization time. At 50 °C, 150 °C, and 220 °C in air, more C-O bonds turned into C=O bonds and C-C bonds were preserved, which enhanced more cyclization reactions in the process of carbonization. Similar results, reported by Chai et al. [52], showed that when a sufficient stabilization time was used for PVA nanofibers, the structure of the carbon nanofibers remained unchanged. The use of a multistage temperature program (50 °C, 150 °C, and 220 °C) was beneficial in preventing the degradation of nanofibers during the carbonization step and thus PVA/PVP-8 had the highest carbon content.

**Figure 5:**
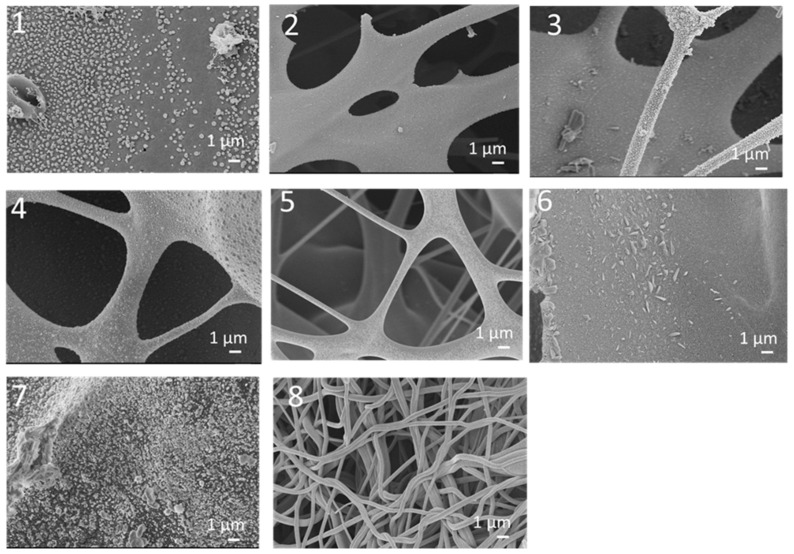
SEM images of centrifugally spun CNFs prepared using PVA/PVP under different stabilization and carbonization conditions.

Figure 5 shows SEM images of PVA/PVP nanofibers carbonized under different conditions. As shown in the SEM images, the morphology of the nanofibers was destroyed and the fibrous structure changed to carbon particles in the first seven processes, and carbonization at high temperatures without a sufficient stabilization time led to the formation of particles. Similar results were reported by Yuniar et al. [53]. The chains of the pyrolyzed and fibrous PVA structure turned into to spherical particles when the carbonization temperature was increased to 300 °C. Wang et al. [54] and Shao et al. [55] also reported that if the stabilization process was not sufficient, then the fibrous and porous structures were destroyed and large weight losses were observed. As shown in the SEM images, in the 8th process, the carbon nanofibers preserved their fibrous structure due to the use of a sufficient stabilization temperature. Applying different stabilization (50, 150 and 220 °C) and carbonization (50, 220, 250 and 800 °C) temperatures was the best way to produce PVA-based carbon nanofibers with a high carbon yield.

### 3.4. Structural and Electrochemical Characterization of Centrifugally Spun B, N, F-Doped Carbon Nanofibers

Nanofibers have unique properties compared to other nanostructured materials, such as a large specific surface area, low density, small diameter, high porosity, and an interpenetrating network, which make them promising materials in energy storage applications [56]. Their large specific surface area provides more active sites and their porous structure decreases the volume expansion that occurs during electrochemical reactions, thus improving utilization and increasing their specific capacity [57].

**Figure 6:**
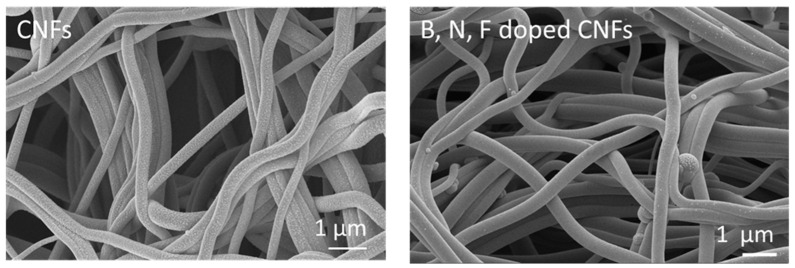
SEM images of centrifugally spun CNFs and B, N, F-doped CNFs.

Figure 6 shows SEM images of centrifugally spun PVA/PVP-derived carbon nanofibers. The average diameter of the PVA/PVP carbon nanofibers was around 300 nm. During carbonization, the average diameter of the nanofibers decreased due to the removal of non-carbon elements from the structure under heat treatment due to their transformation into their gaseous form. The structure of the nanofibers became denser, so the nanofibers shrunk in terms of their diameters and lost weight after carbonization [30].

B, N, F doping has been investigated in a few studies; however, B, N, F doping for anodes in SIBs has not been studied to date. It has been observed that heteroatom doping improves electrochemical properties due to the improvement of defects and improved electronic conductivity [58,59,60]. B, N, F-doped CNFs were prepared in this study by combining centrifugal spinning and heat treatment.

**Figure 7:**
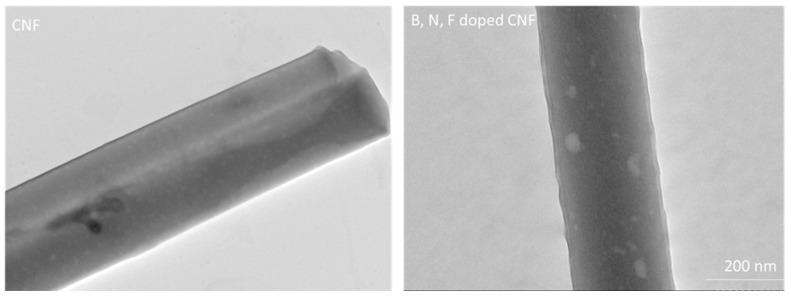
TEM images of centrifugally spun CNFs and B, N, F-doped CNFs.

Figure 6 presents SEM images of B, N, F-doped CNFs. As shown in the SEM images, B, N, F-doped CNFs also have a fibrous morphology, which is useful for electron transport [61,62]. TEM images of CNFs and B, N, F-doped CNFs are presented in Figure 7. As shown in these images, both of them have a long fibrous morphology. The TEM images of B, N, F-doped CNFs display their porous structure, with the surface of the carbon nanofibers becoming rougher, with a high number of defects. The presence of more defects on the surface of fibers obtained through B, N, F doping was also reported by Wang et al. [62].

**Figure 8:**
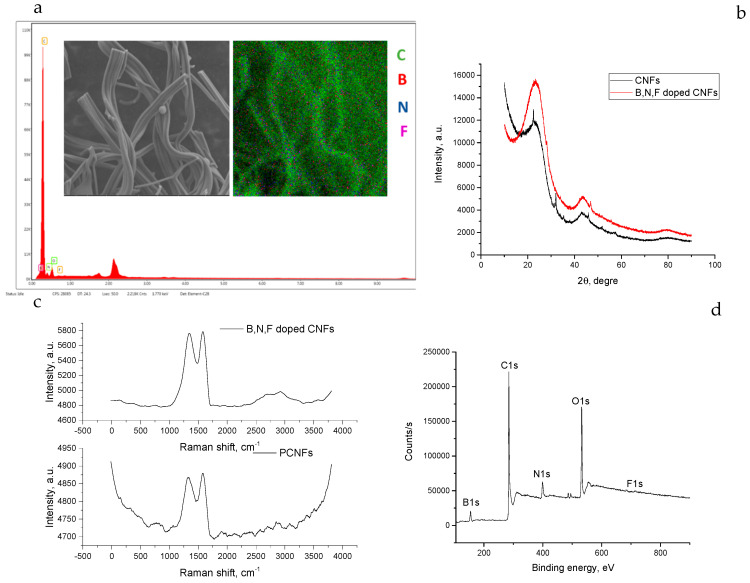
EDX spectra (**a**), XRD pattern (**b**), Raman spectra (**c**), and XPS survey (**d**) results of centrifugally B, N, F-doped CNFs.

The chemical composition of the B, N, F doped CNFs was analyzed by means of EDX spectroscopy (Figure 8a), which confirmed that boron, nitrogen, and fluorine were present in the B, N, F-doped CNFs.

XRD patterns of CNFs and B, N, F-doped CNFs are displayed in Figure 8b. There were two broad diffraction peaks at around ~22° and ~45°, which could be assigned to (002) and (101), which illustrate the amorphous nature of the carbon structure [62,63]. Furthermore, based on the XRD pattern of the B, N, F-doped CNF sample, the intensity of the (002) peak exhibited an increase with the addition of boron, fluorine, and nitrogen elements. The interlayer spacing, d(002), was determined using the Bragg equation, λ = 2dsin θ, where λ is the wavelength of the incident X-ray source from Cu (λ = 1.5406 Å) and θ is the diffraction angle for the peak position [15]. According to the Bragg formula, the graphitic interlayer spacing (d002) values of CNFs and B, F, N-doped CNFs were around 0.39 nm, which is larger than that of natural graphite (0.335 nm) and also demonstrates that doping did not induce major changes in the degree of graphitization [64,65,66]. Raman spectroscopy was also used to further characterize B, N, F-doped CNFs. The D band (defective carbon) and the G band (graphitic carbon) [67] of CNFs and B, F, N-doped CNFs were observed in the Raman spectra, which appeared around ~1345 and ~1585 cm^−1^, respectively. The intensity ratio of I_D_/I_G_ was used to characterize the degree of structural disorder, as well as the degree of defective graphitization and the nature of the graphitization of the carbon material [68]. The I_D_/I_G_ ratios of CNFs and B, N, F-doped CNFs were calculated to be around ~0.9, indicating a disordered structure, which was compatible with the XRD spectra.

In order to study the incorporation of B, N, and F, XPS analysis was also performed and the XPS survey spectra are presented in Figure 8d. Based on the XPS survey, the weight percentages of C, B, N, O, and F were approximately 78%, 2%, 9%, 10%, and 1%, respectively.

Heteroatom doping changes the physical structure of a material and improves its electrochemical properties. For example, doping with nitrogen (N) led to an increase in the numbers of defects and pores in the structure of carbon and increased the active sites for ion storage, which enhanced the capacity and electronic conductivity [69,70]. The interaction and combination of the heteroatoms led to improved physical and chemical properties. Nitrogen is commonly used for co-doping. In a previous study, N/B, N/O, and N/P-Co doping and triple doping not only generated further defect sites in carbon but also promoted its electrochemical activity and enhance its electronic conductivity [71]. Yu et al. [72] prepared sulfur/nitrogen-co-doped carbon nanofibers (SNCNFs) via electrospinning of PAN, followed by carbonization. SNCNFs exhibited a reversible capacity of 170.5 mAhg^−1^, which was much higher than that of nitrogen-doped carbon nanofibers (90 mAhg^−1^). Their electrochemically active –C–S–C– covalent bonds, high specific surface area, and large number of defects with a stable structure contributed to their excellent performance.

**Figure 9:**
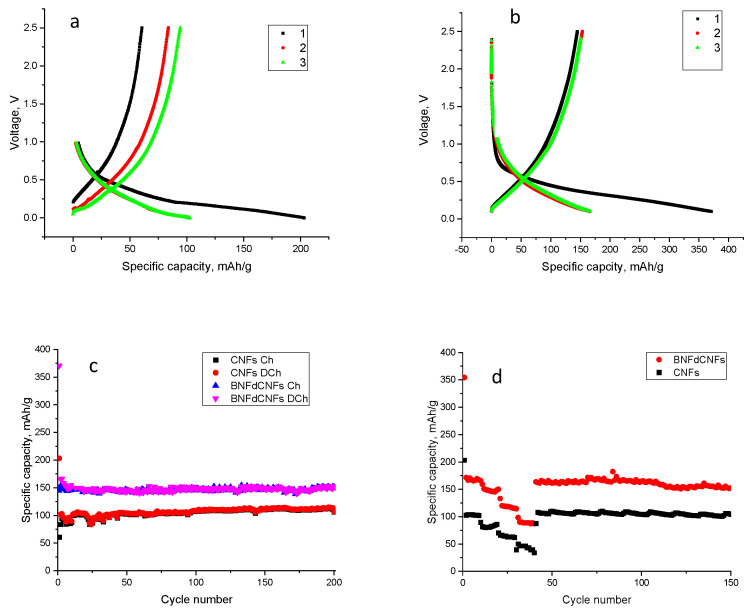
First-cycle discharge charge curves (**a**,**b**) and cycling (**c**) performance at 100 mA/g, as well as the C rate performance (**d**) of centrifugally spun B, N, F-doped carbon nanofibers at current densities of 100, 200, 400, and 800 mA/g.

Figure 9a,b show the discharge/charge voltage profiles of the initial three cycles for CNFs and B, N, F-doped CNFs, respectively. In the first cycle, the discharge and charge capacities were 203 mAh/g and 83 mAh/g, respectively, for CNFs, which led to a low Coulombic efficiency, around 30%, resulting from SEI formation [15]. During the first cycle, the discharge and charge capacities of B, N, F-doped CNFs were 370 and 153 mAhg^−1^, respectively, giving an initial Coulombic efficiency of around 41%. The inevitable formation of an SEI layer was responsible for the 59% capacity loss in the first cycle [73,74]. During the second cycle, the discharge was reduced to 166 mAhg^−1^, with a charge capacity of 150 mAhg^−1^, resulting in a rapid increase in the Coulombic efficiency to 90%, further indicating the good reversibility of the B, F, N-doped CNF electrodes. After 10 cycles, the Coulombic efficiency increased to 99%. Figure 9c displays the cycling performance of CNFs and B, N, F-doped CNFs at a current density of 100 mAg^−1^. It can be seen that the B, N, F-doped CNFs exhibited a high reversible capacity of around 150 mAhg^−1^ in 200 cycles. However, the reversible capacity of CNFs was around 100 mAhg^−1^ in the first 200 cycles. A significant increase in reversible capacity with B, N, F doping was also reported for cathodes in lithium ion batteries [75]. The improvement was ascribed to an enhanced amount of charge carriers, a high number of defects, and a synergetic effect. B, N, F doping also increased the number of active sites and enhanced the electronegativity of electrodes in Zn-air batteries [76].

The rate capability of CNFs and B, N, F-doped CNF electrodes was evaluated at various current densities ranging from 100 to 800 mA g^−1^, as shown in Figure 9d. The average specific capacities for CNF electrodes were around 102, 80, 60, and 40 mAh g^−1^ at current densities of 100, 200, 400, and 800 mA g^−1^, respectively. After bearing large current densities, when the current density returned to 100 mA g^−1^, the discharge specific capacity recovered to around 87 mAh g^−1^. However, the average specific capacities of B, N, F-doped CNF electrodes were around 165, 150, 114, and 88 mAh g^−1^ at current densities of 100, 200, 400, and 800 mA g^−1^, respectively. When the current density returned to 100 mA g^−1^, the discharge specific capacity quickly recovered to 161 mAh g^−1^, indicating its excellent reversibility. In addition, the specific capacity was maintained around 151 mAh g^−1^ after 150 cycles at a current density of 100 mA g^−1^, indicating the long cycling stability of B, N, F-doped CNF electrodes. The good rate capability suggested that B, N, F-doped CNF electrodes are promising anode materials for SIBs. The rate capability of the B, N, F-doped CNFs was superior than that of conventional CNFs because of the semi-ionic C–F bonds, which increased the number of defects in the carbon matrix, and boron acted as an electron acceptor to increase conductivity by shifting the Fermi level to the conduction band [77]. These factors facilitated the fast transfer of Na+ and electrons throughout the electrode.

In this study, centrifugally spun PVA/PVP-based nanofibers have been reported for the first time. Moreover, in order to synthesize carbon nanofibers using environment-friendly polymers, the optimum stabilization and carbonization conditions were determined, considering the nanofibrous structure and the carbon yield. Furthermore, B, N, F-doped CNFs were prepared via heat treatment and used as anodes in SIBs. In Table 3, the electrochemical properties of some reported heteroatom-doped CNF electrodes are presented. Electrospinning has been widely used in previous studies; however, in this study, an easy way to synthesize high-performance anode materials through fast and cost-effective centrifugal spinning has been reported for the first time. Hence, high-performance anodes with many active sites and defects, along with high conductivity, were prepared using a fast and environment-friendly method.

## 4. Conclusions

PVA/PVP-blend nanofibers have been used in many fields and it is important to fabricate PVA/PVP nanofibers using a fast, safe and relatively environmentally-friendly technique. PVA/PVP nanofibers with an average fiber diameter around 700 nm were fabricated via centrifugal spinning. In order to investigate the effect of carbonization conditions on the morphology and carbon yield, different thermal treatment times and temperatures were applied. PVA/PVP-based carbon nanofibers and B, N, F-doped CNFs were fabricated with a combination of centrifugal spinning and thermal treatment. B, N, F-doped CNFs preserved their fibrous structure after heat treatment and delivered a reversible capacity of around 150 mAh g^−1^ at a current density of 100 mA g^−1^. Therefore, we demonstrated that centrifugally spun PVA/PVP-based B, N, F-doped CNFs are a promising anode material candidate for SIBs.

## Figures and Tables

**Table 1:** Viscosity, surface tension, and average fiber diameter.

PVA/PVP	Viscosity	Surface Tension (mN/m)	Average Fiber Diameter (nm)
100/0	395	44	690
75/25	361	41	738
50/50	306	33	747
25/50	254	28	1093
0/100	104	27	2286

**Table 2:** Carbonization conditions for PVA/PVP nanofibers.

Nanofiber	Stabilization	Heating Rate for Stabilization (°C/min)	Carbonization	Heating Rate for Carbonization (°C/min)	Carbon Yield, %
PVA/PVP-1	24 h stabilization at 150 °C, 6 h stabilization at 280 °C	5	carbonization at 800 °C for 2 h	2	6.02
PVA/PVP-2	No stabilization	-	carbonization at 650 °C for 2 h	2	11.61
PVA/PVP-3	24 h stabilization at 150 °C, 6 h stabilization at 280 °C	5	Carbonization at 650 °C for 2 h	2	19.69
PVA/PVP-4	Stabilization in air at 280 °C for 6 h	5	Carbonization at 650 °C for 2 h	2	19.00
PVA/PVP-5	Stabilization in air at 150 °C for 24 h	5	Carbonization at 650 °C for 120 min	2	15.93
PVA/PVP-6	2 h stabilization at 150 °C, 2.5 h stabilization at 280 °C	5	Carbonization at 650 °C for 2 h	2	5.80
PVA/PVP-7	Stabilization at 280 °C for 2.5 h	5	Carbonization at 800 °C for 2 h	2	4.78
PVA/PVP-8	20 °C to 50 °C, 120 min	1	20 °C to 50 °C, 1 h	1	27.16
	50 °C to 150 °C, 16 h	0.5	50 °C to 220 °C, 1 h	1	
			220 °C to 250 °C, 1 h	0.5	
	150 °C to 220 °C, 8 h	0.5	250 °C to 800 °C, 2 h	1	

**Table 3:** Comparison of the performance of the SIBs based on CNF electrodes.

Electrodes	Reversible Capacity	Ref.
N-doped CNFs	100 mAh/g at 100 mA/g	[78]
N-doped CNFs	150 mAh/g at 100 mA/g	[79]
N,F-doped CNFs	150 mAh/g at 100 mA/g	[80]
N-doped CNFs	150 mAh/g at 100 mA/g	[81]
N-doped CNFs	150 mAh/g at 100 mA/g	[72]

## Data Availability

Not applicable.

## References

[1] Winter M., Brodd R.J. (2004). What Are Batteries, Fuel Cells, and Supercapacitors?. Chem. Rev..

[2] Dirican M., Yanilmaz M., Fu K., Lu Y., Kizil H., Zhang X. (2014). Carbon-enhanced electrodeposited SnO2/carbon nanofiber composites as anode for lithium-ion batteries. J. Power Sources.

[3] Yoshio M., Brodd R.J., Kozawa A. (2009). Lithium-Ion Batteries.

[4] Perveen T., Siddiq M., Shahzad N., Ihsan R., Ahmad A., Shahzad M.I. (2020). Prospects in anode materials for sodium ion batteries—A review. Renew. Sustain. Energy Rev..

[5] Kalathil S., Patil S.A., Pant D., Wandelt K. (2018). Microbial Fuel Cells: Electrode Materials. Encyclopedia of Interfacial Chemistry.

[6] Abbas S., Iftikhar M., Ur-Rehman A. (2020). Carbon Anodes for Sodium-Ion Batteries.

[7] Yue L., Zhao H., Wu Z., Liang J., Lu S., Chen G., Gao S., Zhong B., Guo X., Sun X. (2020). Recent advances in electrospun one-dimensional carbon nanofiber structures/heterostructures as anode materials for sodium ion batteries. J. Mater. Chem. A.

[8] Faccini M., Borja G., Boerrigter M., Martín D.M., Crespiera S.M., Vázquez-Campos S., Aubouy L., Amantia D. (2015). Electrospun carbon nanofiber membranes for filtration of nanoparticles from water. J. Nanomater..

[9] Zhao H., Min X., Wu X., Wang H., Liu J., Zhang Z., Huang Z., Liu Y.-G., Fang M. (2017). Microstructure and electrochemical properties of polyacrylonitrile-based carbon micro- and nanofibers fabricated by centrifugal spinning. Chem. Phys. Lett..

[10] Lu Y., Li Y., Zhang S., Xu G., Fu K., Lee H., Zhang X. (2013). Parameter study and characterization for polyacrylonitrile nanofibers fabricated via centrifugal spinning process. Eur. Polym. J..

[11] Lu Y., Yanilmaz M., Chen C., Dirican M., Ge Y., Zhu J., Zhang X. (2015). Centrifugally spun SnO_2_ microfibers composed of interconnected nanoparticles as the anode in sodium-ion batteries. ChemElectroChem.

[12] Atıcı B., Ünlü C.H., Yanilmaz M. (2022). A Review on Centrifugally Spun Fibers and Their Applications. Polym. Rev..

[13] Atıcı B., Ünlü C.H., Yanilmaz M. (2021). A statistical analysis on the influence of process and solution properties on centrifugally spun nanofiber morphology. J. Ind. Text..

[14] Yanilmaz M., Asiri A.M., Zhang X. (2020). Centrifugally spun porous carbon microfibers as interlayer for Li–S batteries. J. Mater. Sci..

[15] Abdolrazzaghian E., Zhu J., Kim J., Yanilmaz M. (2022). MoS_2_-Decorated Graphene@porous Carbon Nanofiber Anodes via Centrifugal Spinning. Nanomaterials.

[16] Lu Y., Fu K., Zhu J., Chen C., Yanilmaz M., Dirican M., Ge Y., Jiang H., Zhang X. (2016). Comparing the structures and sodium storage properties of centrifugally spun SnO2 microfiber anodes with/without chemical vapor deposition. J. Mater. Sci..

[17] Lu Y., Yanilmaz M., Chen C., Ge Y., Dirican M., Zhu J., Li Y., Zhang X. (2015). Lithium-substituted sodium layered transition metal oxide fibers as cathodes for sodium-ion batteries. Energy Storage Mater..

[18] Sada K., Kokado K., Furukawa Y., Kobayashi S., Müllen K. (2015). Polyacrylonitrile (PAN). Encyclopedia of Polymeric Nanomaterials.

[19] Lu Y., Fu K., Zhang S., Li Y., Chen C., Zhu J., Yanilmaz M., Dirican M., Zhang X. (2015). Centrifugal spinning: A novel approach to fabricate porous carbon fibers as binder-free electrodes for electric double-layer capacitors. J. Power Sources.

[20] Flores D., Villarreal J., Lopez J., Alcoutlabi M. (2018). Production of carbon fibers through Forcespinning^®^ for use as anode materials in sodium ion batteries. Mater. Sci. Eng. B.

[21] Al Rai A., Yanilmaz M. (2021). High-performance nanostructured bio-based carbon electrodes for energy storage applications. Cellulose.

[22] Gaaz T.S., Sulong A.B., Akhtar M.N., Kadhum A.A.H., Mohamad A.B., Al-Amiery A.A. (2015). Properties and Applications of Polyvinyl Alcohol, Halloysite Nanotubes and Their Nanocomposites. Molecules.

[23] Patra N., Salerno M., Cernik M., Afshari M. (2017). 22-Electrospun polyvinyl alcohol/pectin composite nanofibers. Electrospun Nanofibers.

[24] Fatema U., Uddin A.J., Uemura K., Gotoh Y. (2011). Fabrication of carbon fibers from electrospun poly(vinyl alcohol) nanofibers. Text. Res. J..

[25] Zhang S.-J., Feng H.-M., Wang J.-P., Yu H.-Q. (2008). Structure evolution and optimization in the fabrication of PVA-based activated carbon fibers. J. Colloid Interface Sci..

[26] Hiremath P., Nuguru K., Agrahari V., Narang A.S., Badawy S.I.F. (2019). Chapter 8-Material Attributes and Their Impact on Wet Granulation Process Performance. Handbook of Pharmaceutical Wet Granulation.

[27] Teodorescu M., Bercea M. (2015). Poly(vinylpyrrolidone)–A Versatile Polymer for Biomedical and Beyond Medical Applications. Polym.-Plast. Technol. Eng..

[28] Nie G., Zhao X., Luan Y., Jiang J., Kou Z., Wang J. (2020). Key issues facing electrospun carbon nanofibers in energy applications: On-going approaches and challenges. Nanoscale.

[29] Zhang J., Sun Y., Zhu J., Kou Z., Hu P., Liu L., Li S., Mu S., Huang Y. (2018). Defect and pyridinic nitrogen engineering of carbon-based metal-free nanomaterial toward oxygen reduction. Nano Energy.

[30] Yanilmaz M., Zhang X. (2015). Polymethylmethacrylate/polyacrylonitrile membranes via centrifugal spinning as separator in Li-ion batteries. Polymers.

[31] Zhang Z., Sun J. (2017). Research on the development of the centrifugal spinning. MATEC Web Conf..

[32] Alipour R., Khorshidi A., Shojaei A.F., Mashayekhi F., Moghaddam M.J.M. (2019). Skin wound healing acceleration by Ag nanoparticles embedded in PVA/PVP/Pectin/Mafenide acetate composite nanofibers. Polym. Test..

[33] Yaseen M., Ammara O., Ahmad W., Shakir M., Subhan S., Subhan F., Khan K., Iqbal M.S. (2021). Preparation of titanium carbide reinforced polymer based composite nanofibers for enhanced humidity sensing. Sens. Actuators A Phys..

[34] Wen P., Hu T.-G., Wen Y., Li K.-E., Qiu W.-P., He Z.-L., Wang H., Wu H. (2021). Development of Nervilia fordii extract-loaded electrospun pva/pvp nanocomposite for antioxidant packaging. Foods.

[35] Bandatang N., Pongsomboon S.-a., Jumpapaeng P., Suwanakood P., Saengsuwan S. (2021). Antimicrobial electrospun nanofiber mats of naoh-hydrolyzed chitosan (HCS)/PVP/PVA incorporated with in-situ synthesized agnps: Fabrication, characterization, and antibacterial activity. Int. J. Biol. Macromol..

[36] Sakurai K., Maegawa T., Takahashi T. (2000). Glass transition temperature of chitosan and miscibility of chitosan/poly(N-vinyl pyrrolidone) blends. Polymer.

[37] Feldstein M.M., Roos A., Chevallier C., Creton C., Dormidontova E.E. (2003). Relation of glass transition temperature to the hydrogen bonding degree and energy in poly(N-vinyl pyrrolidone) blends with hydroxyl-containing plasticizers: 3. Analysis of two glass transition temperatures featured for PVP solutions in liquid poly(ethylene glycol). Polymer.

[38] Ed D.R.L. (1980). Handbook of Water Soluble Gums and Resins.

[39] Abd El-kader M., Abu-Abdeen M. (2012). Thermal and mechanical studies of PVP/2-HEC blend films. Aust. J. Basic Appl. Sci..

[40] Abdullah@Shukry N.A., Ahmad Sekak K., Ahmad M.R., Effendi T.J.B., Ahmad M.R., Yahya M.F. (2014). Characteristics of Electrospun PVA-Aloe vera Nanofibres Produced via Electrospinning. Proceedings of the International Colloquium in Textile Engineering, Fashion, Apparel and Design 2014 (ICTEFAD 2014).

[41] Mano V., Ribeiro E Silva M.E.S., Barbani N., Giusti P. (2004). Binary blends based on poly(N-isopropylacrylamide): Miscibility studies with PVA, PVP, and PAA. J. Appl. Polym. Sci..

[42] Attia G., Abd El-kader M. (2013). Structural, optical and thermal characterization of PVA/2HEC polyblend films. Int. J. Electrochem. Sci..

[43] Saroj A.L., Krishnamoorthi S., Singh R.K. (2017). Structural, thermal and electrical transport behaviour of polymer electrolytes based on PVA and imidazolium based ionic liquid. J. Non-Cryst. Solids.

[44] Yang J.M., Su W.Y., Leu T.L., Yang M.C. (2004). Evaluation of chitosan/PVA blended hydrogel membranes. J. Membr. Sci..

[45] Seok J.Y., Song S.A., Yang I., Woo K., Park S.Y., Park J.H., Kim S., Kim S.S., Yang M. (2020). Hierarchically Porous Carbon Nanofibers with Controllable Porosity Derived from Iodinated Polyvinyl Alcohol for Supercapacitors. Adv. Mater. Interfaces.

[46] Zhang S.-J., Yu H.-Q., Feng H.-M. (2006). PVA-based activated carbon fibers with lotus root-like axially porous structure. Carbon.

[47] Salleh W.N.W., Ismail A.F. (2013). Effect of Stabilization Condition on PEI/PVP-Based Carbon Hollow Fiber Membranes Properties. Sep. Sci. Technol..

[48] Ju J., Kang W., Deng N., Li L., Zhao Y., Ma X., Fan L., Cheng B. (2017). Preparation and characterization of PVA-based carbon nanofibers with honeycomb-like porous structure via electro-blown spinning method. Microporous Mesoporous Mater..

[49] Cai J., Li W., Zhao P., Yu J., Yang Z. (2018). Low-cost and high-performance electrospun carbon nanofiber film anodes. Int. J. Electrochem. Sci..

[50] Peniche C., Zaldívar D., Pazos M., Páz S., Bulay A., Román J.S. (1993). Study of the thermal degradation of poly(N-vinyl-2-pyrrolidone) by thermogravimetry–FTIR. J. Appl. Polym. Sci..

[51] Loría-Bastarrachea M.I., Herrera-Kao W., Cauich-Rodríguez J.V., Cervantes-Uc J.M., Vázquez-Torres H., Ávila-Ortega A. (2011). A TG/FTIR study on the thermal degradation of poly(vinyl pyrrolidone). J. Therm. Anal. Calorim..

[52] Chai S., Zan G., Dong K., Wu T., Wu Q. (2021). Approaching Superfoldable Thickness-Limit Carbon Nanofiber Membranes Transformed from Water-Soluble PVA. Nano Lett..

[53] Yuniar R.A., Widiyastuti W., Setyawan H., Purwaningsih H., Machmudah S., Anggoro D. (2019). Formation of Carbon Fibres From Polymer Poly(vinyl alcohol)/Acetylene Black using Electrospinning Method. IOP Conf. Ser. Mater. Sci. Eng..

[54] Wang P., Zhang D., Ma F., Ou Y., Chen Q.N., Xie S., Li J. (2012). Mesoporous carbon nanofibers with a high surface area electrospun from thermoplastic polyvinylpyrrolidone. Nanoscale.

[55] Shao L., Chung T.-S., Pramoda K.P. (2005). The evolution of physicochemical and transport properties of 6FDA-durene toward carbon membranes; from polymer, intermediate to carbon. Microporous Mesoporous Mater..

[56] Haichao L., Li H., Bubakir M.M., Yang W., Barhoum A., Barhoum A., Bechelany M., Makhlouf A.S.H. (2019). Engineering Nanofibers as Electrode and Membrane Materials for Batteries, Supercapacitors, and Fuel Cells. Handbook of Nanofibers.

[57] Ji L., Lin Z., Alcoutlabi M., Zhang X. (2011). Recent developments in nanostructured anode materials for rechargeable lithium-ion batteries. Energy Environ. Sci..

[58] Bao Y., Huang Y., Song X., Long J., Wang S., Ding L.-X., Wang H. (2018). Heteroatom doping and activation of carbon nanofibers enabling ultrafast and stable sodium storage. Electrochim. Acta.

[59] Chen L.-F., Huang Z.-H., Liang H.-W., Gao H.-L., Yu S.-H. (2014). Three-Dimensional Heteroatom-Doped Carbon Nanofiber Networks Derived from Bacterial Cellulose for Supercapacitors. Adv. Funct. Mater..

[60] Hao R., Lan H., Kuang C., Wang H., Guo L. (2018). Superior potassium storage in chitin-derived natural nitrogen-doped carbon nanofibers. Carbon.

[61] Ma Y., Zhang X., Liang Z., Wang C., Sui Y., Zheng B., Ye Y., Ma W., Zhao Q., Qin C. (2020). B/P/N/O co-doped hierarchical porous carbon nanofiber self-standing film with high volumetric and gravimetric capacitance performances for aqueous supercapacitors. Electrochim. Acta.

[62] Wang Y., Gan R., Zhao S., Ma W., Zhang X., Song Y., Ma C., Shi J. (2022). B, N, F tri-doped lignin-derived carbon nanofibers as an efficient metal-free bifunctional electrocatalyst for ORR and OER in rechargeable liquid/solid-state Zn-air batteries. Appl. Surf. Sci..

[63] Cao L., Zhou X., Li Z., Su K., Cheng B. (2019). Nitrogen and fluorine hybridization state tuning in hierarchical honeycomb-like carbon nanofibers for optimized electrocatalytic ORR in alkaline and acidic electrolytes. J. Power Sources.

[64] Xu Y., Zhang C., Zhou M., Fu Q., Zhao C., Wu M., Lei Y. (2018). Highly nitrogen doped carbon nanofibers with superior rate capability and cyclability for potassium ion batteries. Nat. Commun..

[65] Ding J., Li Z., Cui K., Boyer S., Karpuzov D., Mitlin D. (2016). Heteroatom enhanced sodium ion capacity and rate capability in a hydrogel derived carbon give record performance in a hybrid ion capacitor. Nano Energy.

[66] Sun K., Yang Q., Zheng Y., Zhao G., Zhu Y., Zheng X., Ma G. (2017). High performance symmetric supercapacitor based on sunflower marrow carbon electrode material. Int. J. Electrochem. Sci..

[67] Jian Z., Luo W., Ji X. (2015). Carbon electrodes for K-ion batteries. J. Am. Chem. Soc..

[68] Liang J., Zhao J., Li Y., Lee K.-T., Liu C., Lin H., Cheng Q., Lan Q., Wu L., Tang S. (2017). In situ SiO_2_ etching strategy to prepare rice husk-derived porous carbons for supercapacitor application. J. Taiwan Inst. Chem. Eng..

[69] Lota G., Fic K., Frackowiak E. (2011). Carbon nanotubes and their composites in electrochemical applications. Energy Environ. Sci..

[70] Hulicova-Jurcakova D., Seredych M., Lu G.Q., Bandosz T.J. (2009). Combined Effect of Nitrogen- and Oxygen-Containing Functional Groups of Microporous Activated Carbon on its Electrochemical Performance in Supercapacitors. Adv. Funct. Mater..

[71] Chen W., Wan M., Liu Q., Xiong X., Yu F., Huang Y. (2019). Heteroatom-Doped Carbon Materials: Synthesis, Mechanism, and Application for Sodium-Ion Batteries. Small Methods.

[72] Yu M., Yin Z., Yan G., Wang Z., Guo H., Li G., Liu Y., Li L., Wang J. (2020). Synergy of interlayer expansion and capacitive contribution promoting sodium ion storage in S, N-Doped mesoporous carbon nanofiber. J. Power Sources.

[73] Lin Y., Qiu Z., Li D., Ullah S., Hai Y., Xin H., Liao W., Yang B., Fan H., Xu J. (2018). NiS_2_@CoS_2_ nanocrystals encapsulated in N-doped carbon nanocubes for high performance lithium/sodium ion batteries. Energy Storage Mater..

[74] Wu C., Shen L., Chen S., Jiang Y., Kopold P., van Aken P.A., Maier J., Yu Y. (2018). Top-down synthesis of interconnected two-dimensional carbon/antimony hybrids as advanced anodes for sodium storage. Energy Storage Mater..

[75] Meng Y., Li Y., Xia J., Hu Q., Ke X., Ren G., Zhu F. (2019). F-doped LiFePO@N/B/F-doped carbon as high performance cathode materials for Li-ion batteries. Appl. Surf. Sci..

[76] Yi M., Li N., Lu B., Li L., Zhu Z., Zhang J. (2021). Single-atom Pt decorated in heteroatom (N, B, and F)-doped ReS_2_ Grown on Mo_2_CT_x_ for efficient pH-universal hydrogen evolution reaction and flexible Zn–air batteries. Energy Storage Mater..

[77] Hu Y., Shen L., Wei X., Long Z., Guo X., Qiu X. (2019). One-Pot Synthesis of Novel B, N Co–Doped Carbon Materials for High-Performance Sodium-Ion Batteries. ChemistrySelect.

[78] Chen X., Liu H., Zhou M., Fang G., Zhang H., Cai Z., Zhao X., Xiao L., Liu S., Zhang Y. (2022). Construting sTable 2 × 2 tunnel-structured K_1.28_Ti_8_O_16_@N-doped carbon nanofibers for ultralong cycling sodium-ion batteries. Electrochim. Acta.

[79] Ruan B., Wang J., Shi D., Xu Y., Chou S., Liu H., Wang J. (2015). A phosphorus/N-doped carbon nanofiber composite as an anode material for sodium-ion batteries. J. Mater. Chem. A.

[80] Yan X., Liang S., Shi H., Hu Y., Liu L., Xu Z. (2021). Nitrogen-enriched carbon nanofibers with tunable semi-ionic CF bonds as a stable long cycle anode for sodium-ion batteries. J. Colloid Interface Sci..

[81] Huang Y., Wang L., Lu L., Fan M., Yuan F., Sun B., Qian J., Hao Q., Sun D. (2018). Preparation of bacterial cellulose based nitrogen-doped carbon nanofibers and their applications in the oxygen reduction reaction and sodium–ion battery. New J. Chem..

